# Exudative Retinal Detachments: A Rare Adverse Effect of Topiramate

**DOI:** 10.1002/ccr3.72977

**Published:** 2026-06-15

**Authors:** Sarah Aljefri, Meshal Alzakari, Bailasan Milibari, Doaa Milibari

**Affiliations:** ^1^ Department of Ophthalmology, College of Medicine Imam Mohammad Ibn Saud Islamic University (IMSIU) Riyadh Saudi Arabia; ^2^ College of Medicine Imam Mohammad Ibn Saud Islamic University (IMSIU) Riyadh Saudi Arabia; ^3^ Faculty of Medicine Umm Al‐Qura University Makkah Saudi Arabia; ^4^ King Abdullah Medical City (KAMC) Eye Center Makkah Saudi Arabia

**Keywords:** exudative retinal detachment, idiopathic intracranial hypertension, ocular toxicity, topiramate

## Abstract

Topiramate‐induced multifocal exudative retinal detachment is a rare but potentially vision‐threatening adverse effect. We report a 59‐year‐old woman with idiopathic intracranial hypertension who developed painless bilateral blurry vision and color desaturation 8 days after initiation of topiramate 25 mg twice daily. Fundus examination and spectral‐domain optical coherence tomography demonstrated bilateral multifocal exudative retinal detachment with subretinal fluid. Extensive infectious and inflammatory investigations were unremarkable. Topiramate was discontinued, resulting in complete anatomical resolution of subretinal fluid and improvement of visual acuity to 20/30 in both eyes over 3 months of follow‐up. This case highlights the importance of recognizing this uncommon ocular complication and the potential for complete recovery following prompt drug cessation.

## Key Clinical Message

Topiramate may rarely cause bilateral multifocal exudative retinal detachment even at low therapeutic doses and without angle closure. Early recognition of new visual symptoms and prompt discontinuation of the medication can lead to complete anatomical and visual recovery and prevent permanent vision loss.

## 1 Introduction

Topiramate is a sulfamate‐derived monosaccharide with multiple pharmacological actions, including sodium channel blockade, potassium channel enhancement, carbonic anhydrase inhibition, and stimulation of postsynaptic gamma‐aminobutyric acid (GABA) [1]. Initially approved by the FDA for epilepsy [2], its clinical applications have expanded to include.

Migraine prophylaxis and weight management. It is also prescribed off‐label for bipolar disorder, depression, idiopathic intracranial hypertension, and neuropathic pain [1]. Despite its therapeutic utility, topiramate is associated with several ocular adverse effects. The most common is secondary angle‐closure glaucoma, caused by drug‐induced ciliochoroidal effusion and forward displacement of the lens–iris diaphragm [3, 4]. Other reported complications include acute myopia, ocular hyperemia, scleritis, uveitis, ciliochoroidal effusion, visual field defects, and exudative retinal detachment [3]. Although the incidence of these complications is not well established, acute‐closure glaucoma is estimated to occur in approximately 3 per 100,000 users [5].

While anterior segment complications of topiramate, particularly acute angle closure, are relatively well documented [4], posterior segment involvement is exceptionally rare. Only a limited number of reports have described exudative neurosensory retinal detachment (ERD), macular neurosensory detachment, choroidal detachment, or rhegmatogenous retinal detachment associated with this medication [6, 7, 8, 9, 10, 11, 12, 13, 14]. The precise mechanisms remain speculative.

Given the expanding use of topiramate across medical specialties, awareness of its potential ocular toxicity is essential. Early Identification and prompt discontinuation can prevent irreversible visual loss. To our knowledge, this is the first reported case from Saudi Arabia of topiramate‐induced multifocal ERD.

## 2 Case History/Examination

A 59‐year‐old woman with idiopathic intracranial hypertension underwent lumbo‐peritoneal shunt placement after failing medical therapy. She was then started on topiramate 25 mg twice daily for persistent migraine‐like headaches. Eight days later, she presented with painless bilateral blurry vision and color desaturation. Systemic review was unremarkable, with no history suggestive of infectious or inflammatory diseases, including tuberculosis exposure, high‐risk sexual behavior, skin or genital lesions, joint symptoms or constitutional symptoms. She denied any history of previous ocular inflammation, penetrating ocular trauma, ocular surgeries, or use of topical, local, or systemic corticosteroids.

**FIGURE 1:**
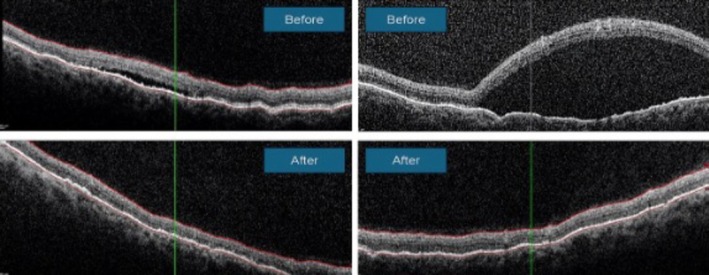
Serial spectral‐domain optical coherence tomography (SD‐OCT) images demonstrating resolution of topiramate‐induced exudative retinal detachment. Top row: SD‐OCT images at presentation showing bilateral subretinal fluid consistent with exudative retinal detachment. Bottom row: SD‐OCT images obtained 3 months after discontinuation of topiramate demonstrating complete resolution of subretinal fluid and restoration of normal retinal contour.

On examination, uncorrected visual acuity was 20/100 in the right eye and 20/50 in the left eye; best‐corrected visual acuity improved to 20/30 and 20/40, respectively. Refraction was +1.25–0.50 × 80 OD and + 1.25–0.75 × 90 OS. Intraocular pressure was 15 mmHg bilaterally. Color vision score was 14/15 in both eyes. No proptosis or pain with eye movements. Slit‐lamp examination revealed clear corneas; the anterior chambers were deep and quiet bilaterally, and a cataract was also noted in both lenses. Fundus examination revealed Paton's lines of optic nerve heads with multifocal ERD in both eyes. Spectral‐domain optical coherence tomography (SD‐OCT) revealed subretinal fluid in both eyes (Figure 1). Fundus autofluorescence photos revealed areas of hyper‐autofluorescence signals that matched the areas of multifocal detachment.

## 3 Differential Diagnosis, Investigations and Treatment

B‐scan showed bilateral ERD with no signs of posterior scleritis, choroidal detachment or intraocular masses. Blood pressure readings were within the normal range, which makes malignant hypertension an unlikely diagnosis in this case. A uveitis workup was conducted to exclude other etiologies, which were inconclusive. Both infectious causes, including syphilis and tuberculosis, and inflammatory conditions such as sarcoidosis and systemic lupus erythematosus were excluded. Also, uveitic masquerade syndrome, such as atypical central serous chorioretinopathy (CSCR), posterior scleritis, and ocular lymphoma, were considered and ruled out.

Based on the clinical findings, the diagnosis of drug‐induced exudative retinal detachment was established, and topiramate was stopped.

## 4 Conclusion and Results (Outcome and Follow‐Up)

The patient was monitored with serial SD‐OCT and FAF imaging. Over 3 months of follow‐up, her vision improved to 20/30 in both eyes with spontaneous subretinal fluid resolution (Figure 1).

Topiramate, even at low doses, can induce reversible multifocal exudative retinal detachment independent of angle‐closure mechanisms. Prompt recognition of new visual symptoms and immediate discontinuation of the medication are essential to ensure full visual recovery. This case expands the spectrum of topiramate‐associated posterior segment toxicity and underscores the importance of clinician awareness and patient education.

## 5 Discussion

This case of bilateral multifocal exudative retinal detachment shortly after topiramate initiation adds to the limited literature describing posterior segment complications of this medication.

While most ophthalmic adverse effects of topiramate involve acute angle‐closure glaucoma and myopic shift, exudative retinal detachment remains extremely uncommon.

Several mechanisms have been proposed to explain topiramate‐induced retinal toxicity. First, carbonic anhydrase inhibition may disturb fluid transport across the retinal pigment epithelium (RPE) and choroid, promoting subretinal fluid accumulation. Second, effects on ion channels and postsynaptic GABA receptors may alter osmotic gradients within the RPE–choroid complex, impairing the RPE pump. Third, an idiosyncratic inflammatory response has been suggested, particularly given the rarity and unpredictability of severe ocular events. Some authors propose that drug‐induced ciliochoroidal effusion may precede secondary RPE dysfunction, ultimately resulting in serous or exudative detachment [3, 6, 7].

Bilateral exudative retinal detachment has a broad differential diagnosis and requires careful and structured clinical evaluation. Inflammatory conditions such as Vogt‐Koyanagi‐Harada (VKH) disease and systemic lupus erythematosus (SLE) are important considerations. VKH typically presents with systemic features, including tinnitus, meningismus, headache, and other neurologic or auditory symptoms; however, none of these were present in our patient. In addition, VKH‐related retinal detachment is more commonly diffuse rather than multifocal, as observed in this case. SLE can also present with bilateral serous retinal detachment, but it is usually accompanied by clear systemic manifestations such as malar rash, arthritis, renal involvement, and elevated inflammatory markers, all of which were absent in this patient's evaluation. Infectious etiologies were excluded through detailed history and appropriate laboratory workup. Malignant hypertension may cause bilateral exudative retinal detachment, often associated with optic disc edema and should always be associated by significantly high blood pressure; both were missing in our presented case. Malignancies, including metastatic disease and intraocular lymphoma, may cause exudative retinal detachment; however, the absence of constitutional symptoms and lack of infiltrative retinal or choroidal lesions made these diagnoses unlikely. Central serous chorioretinopathy (CSCR) can occasionally present bilaterally, but it typically affects young males and is often associated with corticosteroid use, along with stress‐related or type A personality traits; none exist in this case. Given the clear temporal association with initiation of topiramate and the complete anatomical and functional recovery following drug discontinuation, a diagnosis of topiramate‐induced exudative retinal detachment was established. This causality is further strengthened by the rapid resolution after withdrawal, supporting a reversible pharmacologic mechanism rather than a primary structural retinal disorder.

Several related cases have been reported. Dey et al. described patients who developed rhegmatogenous retinal detachment while using topiramate; both had predisposing lattice degeneration, which raises the possibility of coincidence rather than a causal relationship [14]. Dehghani et al. reported a case of massive bilateral choroidal detachment following topiramate use, supporting the theory of choroidal effusion secondary to RPE pump failure [6]. In 2017, Rosenberg et al. described a case of macular neurosensory detachment occurring within days to weeks after topiramate initiation, with complete resolution following drug discontinuation [13]. More recently, Sahin et al. reported a bilateral CSCR‐like picture shortly after topiramate initiation, which also resolved completely after cessation of the medication [15]. Compared with previous reports, our case is notable for its rapid onset within 8 days of starting a low therapeutic dose, its multifocal presentation rather than localized macular involvement, and the absence of angle‐closure, myopic shift, or elevated intraocular pressure. Complete anatomical functional recovery following prompt discontinuation supports a reversible, nonstructural mechanism.

To our knowledge, this represents the first case reported in Saudi Arabia of multifocal exudative retinal detachment associated with topiramate. Recognition of this rare presentation is particularly important in patients treated for idiopathic intracranial hypertension or chronic headache syndromes, where visual changes may otherwise be misattributed to the underlying disease.

Below previously reported cases of retinal detachment or serous/macular detachment temporally associated with topiramate use are summarized in Table 1.

**TABLE 1:** Previously reported cases of retinal or serous detachment associated with topiramate use.

Year	Authors	Presentation	Latency (after topiramate)	Laterality	Remarks
2008	Dey et al. [14]	Two cases of RRD (lattice degeneration) with topiramate use	~12–18 months	Bilateral in one	Possibility of underlying lattice degeneration rather than drug induced RD
2011	Dehghani et al. [6]	Massive bilateral choroidal detachment induced by topiramate	Not specified	Bilateral choroidal effusion	RPE/choroid fluid hypothesis
2017	Rosenberg et al. [13]	Macular neurosensory retinal detachment (two patients)	Days to week after initiation	One bilateral, one unilateral	Resolved on cessation of drug
2022	Sahin et al. [15]	Bilateral central serous‐ chorioretinopathy‐like detachment associated with topiramate	Shortly after initiation	Bilateral	Supports serous detachment resolved on cessation of drug

## Author Contributions

**Sarah Aljefri:** conceptualization, data curation, formal analysis, funding acquisition, investigation, methodology, project administration, resources, software, supervision, validation, visualization, writing – original draft, writing – review and editing. **Meshal Alzakari:** conceptualization, data curation, formal analysis, funding acquisition, investigation, methodology, project administration, resources, software, supervision, validation, visualization, writing – original draft, writing – review and editing. **Bailasan Milibari:** conceptualization, investigation, methodology, resources, software, visualization, writing – original draft. **Doaa Milibari:** conceptualization, data curation, formal analysis, funding acquisition, resources, software, supervision, validation, visualization, writing – original draft, writing – review and editing.

## Funding

The authors have nothing to report.

## Ethics Statement

The study protocol was approved by the Institutional Review Board at King Abdullah Medical City and adhered to the Declaration of Helsinki.

## Consent

Written informed consent was obtained from the patient for publication of the details of their medical case and any accompanying images.

## Conflicts of Interest

The authors declare no conflicts of interest.

## Data Availability

No research data is available outside of the manuscript.
